# The Relationship between Personality Traits, Work–Family Support and Job Satisfaction among Frontline Power Grid Workers

**DOI:** 10.3390/ijerph20032637

**Published:** 2023-02-01

**Authors:** Xiao Zhou, Hualiang Li, Qiru Wang, Chaolin Xiong, Aihua Lin

**Affiliations:** 1 School of Public Health, Sun Yat-sen University, Guangzhou 510080, China; 2 Electric Power Research Institute of Guangdong Power Grid Corporation, Guangzhou 510062, China; 3 School of Health Sciences, Guangzhou Xinhua University, Guangzhou 510520, China

**Keywords:** power grid workers, personality traits, work–family support, job satisfaction

## Abstract

Frontline power grid workers are always facing plenty of stressors such as aerial work and high job demands, which may lead them to be less satisfied with their job. Therefore, this study aims to investigate frontline power grid workers’ job satisfaction (JS) and explore how it can be improved by its relationship with personality traits and work–family support (WFS). Data from 535 frontline power grid workers were collected from two power supply bureaus in Guangdong Province, China. Structural equation modeling (SEM) was adopted to examine the structural relationship between personality traits taken as independent variables, JS as dependent variable, and WFS as mediator. The bootstrap method was used to test the significance of indirect effects. Results suggested the overall job satisfaction of our sample is 3.34 ± 0.55 on a scale ranging from 1 to 5, and significantly correlated with personality traits and WFS. Moreover, the results of SEM and bootstrap indicated that WFS partially mediates the effect of neuroticism on JS and fully mediates the effect of conscientiousness and extraversion on JS. These findings shed light on how personality traits and environmental factors jointly impact JS and highlight the important role of WFS among frontline power grid workers.

## 1. Introduction

With the rapid development of the economy, the demand for energy such as electricity has been increasing rapidly over the past decades. And China, the world’s largest consumer of electricity, consumed 7714 Twh of electricity in 2021, which accounted for 31% of the whole world [1]. Chinese frontline power grid workers have undertaken and contributed a lot. They are mainly responsible for maintaining the power grid that transmits high-voltage electricity and are characterized by exposure to multiple stressors such as aerial work, electric shock, shift work, poor body posture, and outdoor work [2]. However, due to the commercialization of the power industry in recent years, deregulation, privatization, and reductions in welfare programs have resulted in less job security [3], which might lead workers to be less satisfied with their job. However, front-line power grid workers are a relatively minor group, and the literature addressing job satisfaction, turnover intention, as well as the mental health status of this professional category is scarce, and the large majority of publications are concentrated on matters related to exposure to electromagnetic fields [2].

Job satisfaction (JS) is conceptualized as a pleasurable or positive emotional state derived from the appraisal of one’s job or job experiences [4]. It includes cognitive, affective, and behavioral aspects and is also deemed to have multiple applications and repercussions both at work and in people’s daily lives [5]. Previous research has reported its negative association with negative emotions including anxiety, depression, anger, and burnout [6,7], as well as its positive association with marital satisfaction [8], life satisfaction [9], and physical and psychological health [10,11]. Moreover, JS is shown to be positively correlated with numerous organizational outcomes [12,13] such as greater job involvement, better performance, lower levels of turnover intentions, and fewer counterproductive work behaviors. Furthermore, Heimerl [14] believed JS could help promote a sustainable working environment and act as a driver for sustainable development. Given the important role of JS, it is meaningful to explore its antecedents and how they have effects on JS. And personality traits, defined as characteristic patterns of thoughts, feelings, and behaviors over time and across situations [15], were deemed one of the most important ones. Personality traits were believed to influence selection and self-selection into jobs. It would affect how individuals experience work events and work conditions, how they emotionally and behaviorally react, how they recall and appraise the events [16], and ultimately have an effect on the JS. At the same time, a social–cognitive model [17] targeting JS indicated personality traits could affect JS not only directly but also indirectly through multiple mediators, such as environmental supports. And for workers in the power supply industry, the workplace and family are their major living environments, where they can receive the most support. Then, work–family support (WFS) began to attract more and more attention [18,19].

Therefore, the aim of this study was to investigate Chinese frontline power grid workers’ JS and explore its relationship with personality traits and WFS using a structural equation model, and we have detailed and argued each sequence of the model, formulating specific hypotheses.

### 1.1. Relationship between Personality Traits and Job Satisfaction

The present study of JS, which focuses on its antecedents, believes personality is one of the most important ones [20]. And among the numerous theoretical models of personality, the Five-Factor Model is considered the most complete and optimal system to measure personality traits, which are composed of conscientiousness (C), extraversion (E), agreeableness (A), neuroticism (N), and openness (O) [21,22,23].

Conscientiousness has been proven particularly relevant in the organizational context, highlighting its associations with important work outcomes such as JS [24,25]. As conscientious people are achievement-oriented and spend more effort and time in their job, they get better performance and thus have a higher JS [26]. Moreover, previous meta-analyses have also shown a significant positive association between conscientiousness and JS, sometimes even the highest among the four positive ones [20,27].

Extroverted people like to spend more time with their friends and focus more on the quantity and intensity of relationships, and thus they are more likely to be rewarded for interpersonal interactions [20]. When it comes to the workplace, extroverts are more satisfied with their jobs. However, a study on frontline care staff in nursing homes in Sweden suggested extraversion has no effect on JS [28].

Agreeable ones usually care about how surrounding people feel and are more likely to seek harmony with others, and thereby have better interpersonal relationships, which could improve their JS [29]. However, Templer [16] believed cultural context is also an influencing factor, and the correlations between agreeableness and JS were highly variable across studies, especially in Western countries with an individualistic orientation. While in collectivistic societies, agreeableness had a relatively stable positive association with JS.

Due to their negative nature, neurotic individuals would be more likely to select themselves into situations that foster negative affect and thus experience more negative life events than others. When these activities happen in the workplace, they would result in a decline in JS [20]. Meanwhile, previous meta-analyses have shown a significantly negative association between neuroticism and JS, with JS always being the highest among the different traits of the Big Five [26].

Openness is related to scientific and artistic creativity, divergent thinking, low religiosity, and political liberalism. None of these psychological states seem to be closely related to JS [20]. Meanwhile, some researchers believe openness can increase emotions in both positive and negative ways [29], rendering its directional influence on JS unclear. At the same time, many other empirical studies and meta-analyses also support it by proving the relationship between them is not statistically significant [20,26,27]. Thus, only four (conscientiousness, extraversion, agreeableness, and neuroticism) of the Big Five personality traits are included in this study.

Based on this evidence, we proposed Hypothesis 1: extraversion, agreeableness, and conscientiousness are positively related to frontline power grid workers’ JS, but neuroticism is negatively related.

### 1.2. Relationship between Personality Traits and WFS

Work–family support (WFS) is a kind of social support, which mainly focuses on the support from the work and family domain, as well as the interaction of them in a holistic way. Up until now, a large number of studies have suggested that personality traits are stable predictors of social support [30,31,32]. As for the mechanism, Pierce [33] and his colleague believe there are three major paths: (1) people are likely to select and create their social environment to be suitable for their personality traits; (2) personality traits may evoke supportive or unsupportive reactions from others; and (3) personality traits may modify how individuals appraise social support.

In the framework of the Five-Factor Model, conscientious individuals always tend to have a strong work ethic and follow through on the promises that they make. Because of this, their social relationships should be more stable and secure, given their dependability [34]. People would prefer to work with them and be satisfied with their relationship, so they are always willing to offer support when stressors arise. Agreeable ones are always described as having modesty, altruism, and straightforwardness. Theoretically, these tendencies may facilitate building a more extensive social support network, which may provide the basis for seeking support [35], and thus they would have a higher probability of receiving support when facing stressors. Extraverted individuals enjoy being around others and typically have a wide circle of friends. As such, when they find themselves in stressful situations, they have a larger number of individuals to turn to for support [34]. And these tendencies were believed to facilitate better coping with stress, and result in a lower need for social support, which ultimately increased the perceived availability of social support [35]. Neurotic individuals are seen as easily irritated or tense and might be difficult to interact with. Moreover, they are less likely to perceive other people as being supportive, and as such, they would receive less support from members of their social network [34]. Barańczuk [35] also believed these characteristics may lead to a greater need for social support, which could decrease the perceived availability of social support.

Drawing on the evidence reviewed, we advanced Hypothesis 2: conscientiousness, extraversion, and agreeableness are positively related to WFS, while neuroticism is negatively related.

### 1.3. Relationship between WFS and Job Satisfaction

In recent years, more and more women have chosen to work outside and equally undertake the pressure of taking care of the family with their husbands [36]. Thus, the interference and spillover would occur more often between the work and family domains, especially in China with a collectivist culture. Then, work–family support began to attract more and more attention. Prior studies have found positive correlations between social support and JS in different occupations [37,38], as social support could help to obtain feedback and suggestions and may help relieve work stress, bring strength and motivation to persist, and ultimately lead to JS [39]. And from the perspective of the work–family interface, work support and family support are both deemed to be vital antecedents of JS [40,41].

Work support has multiple resources such as organizations, supervisors, and co-workers. Researchers believed that when employees accept and perceive support from colleagues and leaders in the organization, they tend to respond by developing loyalty to the organization and demonstrating more passion for their work. Conversely, if employees do not receive the support described above, they may reduce their dependence on the organization and show dissatisfaction with their work [38,42]. Family support is relatively simple and mainly refers to the support from the spouse, children, and other family members. As for its association with JS, Zhang [43] and Siu [44] believed family support can provide buffering effects such that employees can focus more on job-related tasks when necessary and perform better as a result, which ultimately leads to a higher JS. Chan [40] also believes family support is a contextual resource that can facilitate JS.

Moreover, according to work–family enrichment theory [45], work support and family support can facilitate each other, which means an employee who receives support from an organization or from family can transfer from one domain to another, leading to a decrease in work–family conflict and contributing to the worker’s well-being such as JS. An empirical study [46] on public-sector employees also agreed with this.

Based on this evidence, we proposed Hypothesis 3: WFS is positively related to JS.

## 2. Materials and Methods

### 2.1. Setting and Participants

From January to June 2019, this cross-sectional study was conducted at a power supply company in Guangdong Province, China. By then, the company served 20 cities with electricity and thus had 20 power supply bureaus, each with a similar organizational structure.

To facilitate data collection, a cluster sampling method was applied to select the participants. Two bureaus were randomly selected from the company. Workers were invited to use Wenjuanxing, a popular online survey platform in China, to complete the electric questionnaires in a face-to-face interaction with the guidance of enumerators. And the purpose of the study was explained at the beginning. The questionnaire was related to work characteristics, and thus limited to subjects currently working. New recruits (working less than half a year), retired employees, and those on long-term leave were excluded. Initially, a total of 685 workers were recruited for the study. Subjects with missing data or invalid data were excluded from the analysis, resulting in a final study sample of 535 (78.1%) individuals.

### 2.2. Measures

#### 2.2.1. Personality Traits

Personality traits were assessed using the Chinese version of the 60-item NEO Five-Factor Inventory (NEO-FFI) [22,47], which provides a concise measure of the five main personality dimensions proposed by the Five-Factor Model, namely, neuroticism (N), extraversion (E), openness to experience (O), agreeableness (A), and conscientiousness (C), and each scale was assessed with 12 items. Participants were asked for self-ratings on the items using a 5-point Likert scale ranging from 1 (strongly disagree) to 5 (strongly agree). The internal reliabilities (Cronbach’s *α*) in the present study were 0.83, 0.72, 0.69, and 0.82, respectively, for N, E, A, and C. Confirmatory factor analysis was used to measure the validity of the scale, and the values of the indicators can be seen in Table 1.

#### 2.2.2. Work-Family Support

Work–family support was measured by the 30-item Work–Family Support Scale (WFSS), conducted by Li Yongxin (2009) [18]. The scale consisted of four domains: organizational support (OS, 10 items), leadership support (LES, 10 items), emotion support (ES, 6 items), and instrumental support (IS, 4 items). Participants were asked for self-ratings on the items using a 5-point Likert scale ranging from 1 (strongly disagree) to 5 (strongly agree). The internal reliabilities (Cronbach’s *α*) in the present study were 0.89, 0.93, 0.85, and 0.82, respectively, for OS, LES, ES, and IS. The results of the confirmatory factor analysis can be seen in Table 1.

#### 2.2.3. Job Satisfaction

Job satisfaction was assessed by the Chinese version of the Index of Organizational Reactions (IOR) [48,49], which consists of 42 items and eight domains: supervision (SV, 6 items), kind of work (KW, 6 items), company identification (CI, 5 items), amount of work (AW, 4 items), physical surrounding (PS, 6 items), financial rewards (FR, 5 items), career future (CF, 5 items), and co-workers (CW, 5 items). The internal reliabilities (Cronbach’s *α*) in the present study were 0.84, 0.84, 0.74, 0.68, 0.86, 0.82, 0.76, and 0.66, respectively, for SV, KW, CI, AW, PS, FR, CF, and CW. The results of the confirmatory factor analysis can be seen in Table 1.

### 2.3. Statistical Analysis

Descriptive statistics and correlation analyses were calculated with SPSS (IBM, released 2019, SPSS Statistics for Windows Version 26.0, Armonk, New York). The mean and standard deviation were used to describe the score of each dimension scale, and *t*(*t*’)- test or analysis of variance (ANOVA) was used to test the difference between groups of variables. Pearson’s r correlations were calculated to explore the association between variables. Skewness and kurtosis were used to test the normality of our main variables. For the skewness index, absolute values greater than 3.0 are extreme. For Kurtosis, absolute values higher than 10.0 suggest a problem, and values higher than 20.0 are extreme [50]. The skewness of study variables ranged from 0.040 to 0.697, and the kurtosis statistics ranged from 0.042 to 1.320. Confirmatory factor analysis was conducted to verify the construct validity of questionnaires.

The main hypotheses were tested with Amos (IBM, released 2016, SPSS Amos for Windows Version 24.0, Armonk, NY, USA) to conduct structural equation modeling (SEM). As previous studies indicated that age [51] and salary [52] have a significant influence on JS, they were incorporated into the model as control variables. For model fit evaluation, absolute and comparative fit indices, including *χ*^2^/df, the root mean squared error of approximation (RMSEA), comparative fit index (CFI), incremental fit index (IFI), Tucker–Lewis index (TLI), adjusted goodness of fit index (AGFI), and standardized root mean square residual (SRMR), were used to evaluate whether the data fit the hypothesized model.

In addition, bootstrap analysis was used to test the significance of indirect effects. The number of bootstrap samples was set at 5000, and a 95% confidence interval (CI) with a bias-corrected percentile method was used. If zero was excluded, the indirect effect would be deemed statistically significant. Moreover, Keith [53] explained the sizes of beta coefficients as: less than 0.05 means too small to be meaningful; 0.05 to 0.10, small but meaningful; 0.10 to 0.25, moderate; and greater than 0.25, large.

## 3. Results

### 3.1. Descriptive Statistics

Table 2 provides the sociodemographic characteristics and scores of the main variables in our sample. Among the total of 535 valid samples, most respondents were male (93.10%), married (79.30%), worked more than 10 years (71.03%), and worked as staff other than a leader (66.40%). About half of them were aged from 41 to 60 (50.50%) and were highly educated, with at least a university education experience (42.10%). As for salary, most of them (41.10%) earned a monthly household income between RMB 4000 and RMB 6000. The descriptions of the scores were as follows: JS (3.34 ± 0.55), WFSS (14.17 ± 2.42), extraversion (40.08 ± 5.57), agreeableness (45.35 ± 4.7), conscientiousness (44.9 ± 5.68), neuroticism (29.79 ± 6.97), and openness (37.6 ± 3.86). And for JS, differences between age group (*p* = 0.009), marital status (*p* < 0.001), position (*p* = 0.022), working years (*p* = 0.013), and salary groups (*p* < 0.001) were statistically significant. More details can be seen in Table 2.

### 3.2. Correlation Analysis

A correlation matrix was used to show the Pearson correlations between all the variables, and all of them were statistically significant with *p* less than 0.01. Bivariate correlations indicated that neuroticism was negatively correlated with all dimensions of WFS and JS, while the other three personality traits (extraversion, agreeableness, and conscientiousness) were positively correlated. Besides, WFS and JS were also positively correlated. More details can be seen in Table 3.

### 3.3. Structural Model

**Figure 1:**
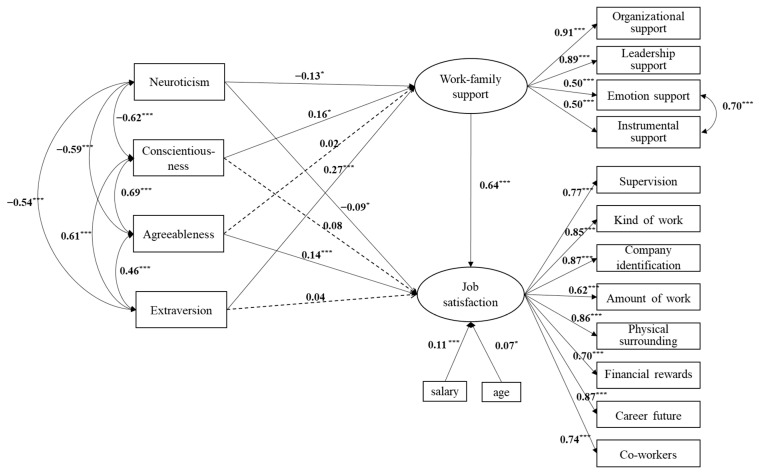
Structural model with standardized path loadings. Note: solid line = path differing significantly from 0; dashed line = path not differing significantly from 0. * *p* < 0.05, *** *p* < 0.001.

Based on hypotheses 1 to 3 and considering the control variables of age and salary, we constructed the initial model. However, the fit indices were not good at first, and according to the modification indices (MI), we added the correlation of the residuals of emotion support and instrumental support in WFS, and then the modified model (Figure 1) showed a relatively better model fit. More details can be seen in Table 1.

For personality traits and WFS, neuroticism (*β* = −0.130, *p* < 0.05), conscientiousness (*β* = 0.16, *p* < 0.05), and extroversion (*β* = 0.27, *p* < 0.001) were all found to have a significant influence on WFS. These results indicated that frontline power grid workers with low neuroticism, high extroversion, and conscientiousness are more likely to experience WFS. For personality traits and JS, neuroticism (*β* = −0.09, *p* < 0.05) and agreeableness (*β* = 0.14, *p* < 0.001) were found to have a significant influence on JS and indicated workers with low neuroticism and high agreeableness were more likely to experience JS. Moreover, WFS (*β* = 0.64, *p* < 0.001) had a positive influence on JS and indicated workers with higher WFS tend to be more satisfied with their job. The control variables, age (*β* = 0.07, *p* < 0.05) and salary (*β* = 0.11, *p* < 0.001), were both found to have significant relationships with JS.

The result of bootstrap analysis suggested WFS partially mediates the effect of neuroticism on JS and fully mediates the effect of conscientiousness and extraversion on JS. However, the relationship between agreeableness and WFS had no statistical significance, so WFS was not a mediator between them. More details can be seen in Table 4.

## 4. Discussion

To our knowledge, this is the first study to explore frontline power grid workers’ JS in relation to personality traits and WFS with a quantitative method, especially in a Chinese context. And we found a slightly lower overall JS (3.34 ± 0.55) than managers (3.62 ± 0.50) [54] and temporary (3.70 ± 0.52) as well as permanent employees (3.44 ± 0.53) from other occupations [55], which indicated JS among frontline power grid workers deserves more attention to be improved. And more job resources and strategies targeting personality traits and work–family support should be highly considered.

In the SEM analysis, we found a close linkage among personality traits (WFS and JS) in frontline power grid workers. As for personality traits and JS, all four personality traits had a moderate total effect on JS, among which extroversion affected the most and was followed by conscientiousness, neuroticism, and agreeableness. And more specifically, we found individuals high in extroversion, conscientiousness, agreeableness, and low in neuroticism were more likely to be satisfied with their job, which was in line with Templer [16] and Hyongdong Kim [29]. However, the relationship between personality traits and JS was not uniform across studies [20,56]. Zhai [57] found that only extraversion was an independent predictor of JS among the five, as he believed the relationship with others in the workplace was much more important. And extroverted people would always have good relationships because of their sociable, talkative, and assertive characteristics, and thus have a higher JS. The discrepancy between results may be due to the sample’s occupation. Törnroos [56] found different occupations have their own average level of personality traits, and the fitness of an individual’s and their occupation’s personality traits would have a significant effect on their JS. Some researchers [58,59] also agreed with it and emphasized the concept of person–organization fit, which describes the congruence between an individual and their organization. Zhai [57] conducted a survey on white-collar employees, whose work is basically non-manual, and for them, communication as well as relationships with others played a much more important role than for front-line workers.

As for personality traits and WFS, the results were mostly consistent with previous studies indicating individuals high in extroversion, conscientiousness and low in neuroticism were more likely to get WFS. However, in our research, the effect of agreeableness on WFS was not statistically significant. This discrepancy tends to suggest two alternative explanations: On the one hand, it may be due to the fit of individuals and their occupation. The person–environment fit theory believes people tend to prefer and thrive in environments that are “compatible” with their characteristics [60] and thus produce more positive outcomes. Just like personality traits and JS [56], the association between an individual’s personality and WFS in different occupations was also partly subject to the average level of personality traits in that occupation. On the other hand, personality traits were not isolated from each other. Swickert [34] believed except for openness is generally orthogonal with respect to the other four personality traits, neuroticism, extraversion, conscientiousness, and agreeableness are all moderately correlated with each other. This collinearity could lead to competition regarding the amount of variance they can account for in the perceived availability of support, which can explain the non-statistically significant association in our study.

Meanwhile, workers experiencing a higher level of WFS were inclined to be more satisfied with their jobs. And social exchange theory believes that when employees perceive that their organizations and families are offering help, they will feel more supportive and more obligated to reciprocate by feeling more satisfied with their family and job [61]. Morman [62] also believed relationship quality, both at home and in the workplace, is essential in helping to mitigate the negative effects of job stress on JS. The bootstrap analysis also proved WFS plays an important mediating role in the relationship between personality traits and JS. More specifically, the results suggested WFS partially mediates the effect of neuroticism on JS, indicating neuroticism affects JS not only directly but indirectly through WFS. At the same time, WFS fully mediated the effect of conscientiousness and extraversion on JS, indicating the correlation between conscientiousness/extraversion and JS was due to the existence of WFS, as they have no direct effect on JS, but only an indirect effect through WFS.

Though personality traits and WFS are both deemed vital predictors of JS, interventions targeting WFS are more practicable compared to personality traits, as personality traits are generally seen as relatively stable characteristics across time and culture and are most affected by heredity. Though there are some longitudinal studies [30,63] that have reported the small effect of environmental factors (e.g., social support) on personality traits, it will be a long-term process to understand the effects. Moreover, Asendorpf [64] believed social support can only predict surface characteristics (global self-worth, perceived peer acceptance, and loneliness) other than core characteristics (Big Five personality traits). Therefore, in future interventions on JS among frontline power grid workers, the role of work–family support should be emphasized. Especially for neurotic, conscientious, and extroverted workers, the establishment of an effective support system can improve their JS.

As with any research, this study was limited in some ways. First, all of the data in this study were collected through a self-reported questionnaire, which may be subject to reporting bias. Multimethod-multimodal approaches such as integrating the use of structured or semi-structured interviews may be considered in future studies. Second, the sample of this study was limited to frontline power grid workers and was collected through cluster sampling, which both might limit the generalization of the results in our study. Therefore, future studies may try to expand the target population and improve the validity of the current research conclusions. Third, as this study was cross-sectional, we could neither prove causality nor confirm whether these associations would change across time. Longitudinal studies would be valuable in the future. Fourth, front-line power grid workers are a relatively minor group and lack more information and research. Meanwhile, we are also missing some basic information such as the health status of this occupational group, and future studies should collect more information to conduct more comprehensive studies. At last, the full version scales were used in this study to obtain more comprehensive data, and while this may result in a heavy respondent burden, the shortened version scale could be considered in future studies.

## 5. Conclusions

In general, this study investigated the personality traits (WFS and JS) as well as their relationship with a representative sample of frontline power grid workers in Guangdong, China. A slightly lower overall JS was found, which indicated more strategies targeting its antecedents should be taken into consideration. Meanwhile, this study revealed how personality traits and WFS jointly impact JS and highlighted the important mediating role of WFS, and thus it is highly recommended to establish an effective support system in both the work and family domains to better improve workers’ JS, especially for neurotic ones. Moreover, the results suggest that, in relation to the recruitment process, conscientious, extraverted, and agreeable workers are more suitable for front-line power grid operations compared with neurotic ones. And this may be used to increase personal resources and be useful to company managers in developing strategies targeting personality traits in cultivating WFS for JS promotion.

## Figures and Tables

**Table 1:** Model fit indicators.

Indicators	Reference	Measurement Models						Structural Model	
		N	C	E	A	WFS	JS	Initial Model	Modification Model
*χ*^2^/df	<5.00	3.581	3.010	4.921	3.546	3.239	2.727	7.166	4.403
RMSEA	<0.08	0.070	0.061	0.086	0.069	0.065	0.057	0.107	0.080
CFI	>0.90	0.909	0.924	0.810	0.843	0.911	0.874	0.876	0.932
IFI	>0.90	0.909	0.924	0.810	0.843	0.911	0.874	0.876	0.932
NNFI(TLI)	>0.90	0.889	0.908	0.768	0.809	0.903	0.862	0.848	0.916
AGFI	>0.80	0.911	0.925	0.89	0.905	0.826	0.800	0.786	0.858
SRMR	<0.10	0.053	0.048	0.065	0.059	0.050	0.053	0.082	0.076

Note: N = neuroticism, E = extraversion, O = openness, A = agreeableness, C = conscientiousness, WFS = work–family support, JS = job satisfaction, RMSEA = root mean squared error of approximation, CFI = comparative fit index, IFI = incremental fit index, TLI = Tucker–Lewis index, AGFI = adjusted goodness of fit index, SRMR = standardized root mean square residual.

**Table 2:** Demographic characteristics and scores of the participants (mean ± standard deviation).

		No.	%	NEO Five-Factor Inventory					WFS	JS
				N	E	O	A	C		
Total		535	100.00	29.79 ± 6.97	40.08 ± 5.57	37.60 ± 3.86	45.35 ± 4.7	44.9 ± 5.68	14.17 ± 2.42	3.34 ± 0.55
Sex										
	female	37	6.90	31.81 ± 6.90	37.76 ± 5.48	37.14 ± 3.72	44.73 ± 4.37	44.73 ± 6.45	13.82 ± 2.19	3.18 ± 0.57
	male	498	93.10	29.64 ± 6.96	40.25 ± 5.54	37.63 ± 3.88	45.40 ± 4.72	44.91 ± 5.63	14.20 ± 2.43	3.35 ± 0.55
	*t*(*t*’)			1.828	−2.641	−0.755	−0.836	−0.184	−0.905	−1.759
	*p*			0.068	**0.009**	0.450	0.403	0.854	0.366	0.079
Age group, year										
	20–40	185	34.60	30.36 ± 7.50	39.55 ± 6.33	37.88 ± 4.07	45.47 ± 4.63	44.85 ± 6.16	14.00 ± 2.45	3.24 ± 0.55
	41–60	270	50.50	29.61 ± 6.79	40.30 ± 5.00	37.41 ± 3.82	45.27 ± 4.56	44.80 ± 5.24	14.24 ± 2.36	3.40 ± 0.54
	>60	80	15.00	29.11 ± 6.29	40.55 ± 5.49	37.57 ± 3.49	45.35 ± 5.33	45.31 ± 5.99	14.33 ± 2.53	3.33 ± 0.56
	*F*			1.081	1.324	0.782	0.095	0.256	0.733	4.700
	*p*			0.340	0.267	0.458	0.909	0.774	0.481	**0.009**
Education										
	high school or below	310	57.90	30.24 ± 6.25	40.01 ± 5.00	37.46 ± 3.80	44.86 ± 4.45	44.19 ± 5.09	14.04 ± 2.40	3.32 ± 0.51
	college or above	225	42.10	29.18 ± 7.83	40.17 ± 6.27	37.78 ± 3.95	46.04 ± 4.95	45.86 ± 6.29	14.35 ± 2.43	3.36 ± 0.60
	*t*(*t*’)			1.668	−0.330	−0.939	−2.880	−3.275	−1.480	−0.918
	*p*			0.096	0.742	0.348	**0.004**	**0.001**	0.140	0.359
Marital status										
	single	111	20.70	31.61 ± 7.28	38.23 ± 6.36	37.29 ± 3.81	45.40 ± 4.65	43.93 ± 6.22	13.65 ± 2.51	3.12 ± 0.56
	married	424	79.30	29.32 ± 6.82	40.56 ± 5.24	37.68 ± 3.88	45.34 ± 4.72	45.15 ± 5.51	14.31 ± 2.37	3.39 ± 0.53
	*t*(*t*’)			3.113	−3.547	−0.949	0.108	−2.021	−2.541	−4.800
	*p*			**0.002**	**0.001**	0.343	0.914	**0.044**	**0.011**	**<0.001**
Position										
	staff	355	66.40	30.64 ± 6.57	39.61 ± 5.18	37.77 ± 3.94	44.74 ± 4.51	44.03 ± 5.28	14.14 ± 2.36	3.28 ± 0.52
	leader	180	33.60	28.12 ± 7.45	41.00 ± 6.17	37.27 ± 3.71	46.56 ± 4.84	46.61 ± 6.07	14.23 ± 2.52	3.45 ± 0.59
	*t*(*t*’)			3.838	−2.596	1.414	−4.302	−4.862	−0.412	−3.416
	*p*			**<0.001**	**0.010**	0.158	**<0.001**	**<0.001**	0.681	**0.001**
Working years										
	<10	155	28.97	29.97 ± 7.65	39.32 ± 6.68	37.61 ± 4.00	45.81 ± 4.82	44.85 ± 6.32	14.24 ± 2.42	3.24 ± 0.57
	≥10	380	71.03	29.72 ± 6.69	40.38 ± 5.03	37.59 ± 3.81	45.17 ± 4.64	44.91 ± 5.41	14.14 ± 2.42	3.37 ± 0.54
	*t*(*t*’)			0.384	−1.784	0.056	1.426	−0.114	0.447	−2.503
	*p*			0.701	0.076	0.955	0.154	0.910	0.655	**0.013**
Salary (RMB, yuan)										
	≤4000	40	7.50	30.75 ± 6.88	40.10 ± 6.02	38.00 ± 4.83	45.75 ± 4.85	44.43 ± 5.06	13.90 ± 2.48	3.12 ± 0.56
	4001–6000	220	41.10	31.14 ± 6.28	39.41 ± 5.30	37.62 ± 3.93	44.51 ± 4.42	43.65 ± 5.16	13.75 ± 2.32	3.20 ± 0.52
	6001–8000	155	29.00	29.65 ± 6.58	39.76 ± 5.06	37.34 ± 3.65	45.31 ± 4.58	44.99 ± 5.31	14.39 ± 2.35	3.39 ± 0.48
	8001–10,000	69	12.90	28.25 ± 7.42	40.13 ± 5.78	37.83 ± 3.62	45.91 ± 4.67	45.54 ± 5.89	14.26 ± 2.44	3.44 ± 0.53
	≥10,000	51	9.50	25.78 ± 8.56	43.80 ± 6.29	37.65 ± 3.77	48.04 ± 5.18	49.49 ± 6.69	15.43 ± 2.50	3.76 ± 0.61
	*F*			7.671	6.910	0.341	6.496	12.228	5.846	14.914
	*p*			**<0.001**	**<0.001**	0.850	**<0.001**	**<0.001**	**<0.001**	**<0.001**

Note: N = neuroticism, E = extraversion, O = openness, A = agreeableness, C = conscientiousness, WFS = work–family support, JS = job satisfaction.

**Table 3:** Correlation matrix between main variables.

		Personality Traits				Work–Family Support					Index of Organizational Reactions								
		N	E	A	C	OS	LES	ES	IS	Total	SV	KW	CI	AW	PS	FR	CF	CW	Total
Personalities																			
	Neuroticism	(0.831)																	
	Extraversion	−0.538 **	(0.716)																
	Agreeableness	−0.594 **	0.465 **	(0.692)															
	Conscientiousness	−0.622 **	0.606 **	0.692 **	(0.815)														
Work–Family Support Scale																			
	Organizational support	−0.329 **	0.372 **	0.264 **	0.340 **	(0.892)													
	Leadership support	−0.351 **	0.386 **	0.309 **	0.376 **	0.810 **	(0.925)												
	Emotion support	−0.243 **	0.454 **	0.317 **	0.397 **	0.408 **	0.469 **	(0.851)											
	Instrumental support	−0.274 **	0.398 **	0.309 **	0.365 **	0.412 **	0.459 **	0.775 **	(0.818)										
	Total	−0.369 **	0.485 **	0.358 **	0.444 **	0.818 **	0.850 **	0.787 **	0.788 **	(0.947)									
Index of Organizational Reactions																			
	Supervision	−0.431 **	0.375 **	0.404 **	0.443 **	0.591 **	0.621 **	0.284 **	0.287 **	0.559 **	(0.842)								
	Kind of work	−0.432 **	0.476 **	0.392 **	0.493 **	0.575 **	0.547 **	0.405 **	0.398 **	0.596 **	0.670 **	(0.841)							
	Company identification	−0.408 **	0.392 **	0.404 **	0.431 **	0.643 **	0.589 **	0.332 **	0.328 **	0.593 **	0.691 **	0.745 **	(0.737)						
	Amount of work	−0.359 **	0.308 **	0.272 **	0.315 **	0.513 **	0.454 **	0.283 **	0.286 **	0.471 **	0.445 **	0.540 **	0.560 **	(0.681)					
	Physical surrounding	−0.401 **	0.436 **	0.429 **	0.433 **	0.620 **	0.580 **	0.391 **	0.393 **	0.611 **	0.620 **	0.745 **	0.745 **	0.589 **	(0.862)				
	Financial rewards	−0.322 **	0.251 **	0.293 **	0.350 **	0.510 **	0.451 **	0.262 **	0.228 **	0.452 **	0.542 **	0.566 **	0.658 **	0.402 **	0.625 **	(0.823)			
	Career future	−0.464 **	0.449 **	0.408 **	0.486 **	0.611 **	0.587 **	0.362 **	0.389 **	0.605 **	0.670 **	0.756 **	0.775 **	0.509 **	0.752 **	0.656 **	(0.763)		
	Co-workers	−0.428 **	0.412 **	0.493 **	0.477 **	0.507 **	0.485 **	0.365 **	0.390 **	0.534 **	0.621 **	0.645 **	0.637 **	0.507 **	0.678 **	0.437 **	0.630 **	(0.661)	
	Total	−0.493 **	0.473 **	0.470 **	0.523 **	0.698 **	0.662 **	0.408 **	0.409 **	0.675 **	0.815 **	0.873 **	0.887 **	0.669 **	0.883 **	0.759 **	0.880 **	0.770 **	(0.956)
	Skewness	0.118	−0.040	0.160	0.145	−0.312	−0.503	−0.697	−0.605	−0.284	−0.390	−0.149	−0.143	−0.594	−0.285	−0.095	−0.188	−0.048	−0.036
	Kurtosis	0.197	0.731	−0.042	0.626	−0.243	0.256	10.320	10.317	0.427	−0.071	0.184	0.309	0.197	0.319	−0.133	0.445	0.469	0.371

Note: ** *p*< 0.01 (two-tailed), Cronbach’s *α* coefficients are displayed on the main diagonal.

**Table 4:** Bootstrap analysis (*N* = 5000).

	Path Way	Estimate	SE	Lower 95%CI	Upper 95%CI	*p*
Direct effect						
	Extraversion-JS	0.038	0.045	−0.051	0.128	0.382
	Agreeableness-JS	0.143	0.041	0.063	0.225	<0.001
	Conscientiousness-JS	0.084	0.045	−0.003	0.171	0.061
	Neuroticism-JS	−0.085	0.039	−0.160	−0.010	0.026
	WFS-JS	0.642	0.033	0.572	0.702	0.001
Indirect effect						
	Extraversion-WFS-JS	0.170	0.046	0.084	0.264	0.001
	Agreeableness-WFS-JS	0.014	0.040	−0.065	0.092	0.737
	Conscientiousness-WFS-JS	0.100	0.046	0.011	0.190	0.029
	Neuroticism-WFS-JS	−0.084	0.040	−0.165	−0.009	0.028
Total effect						
	Extraversion-JS	0.208	0.058	0.092	0.319	<0.001
	Agreeableness-JS	0.157	0.056	0.049	0.266	0.005
	Conscientiousness-JS	0.184	0.060	0.065	0.297	0.004
	Neuroticism-JS	−0.169	0.052	−0.267	−0.067	0.003

Note: WFS = work–family support, JS = job satisfaction, SE = standard error, CI = confidence interval.

## Data Availability

All scales used for the study are available from the corresponding author upon reasonable request. The data are not publicly available due to privacy restrictions.
